# Stress Distribution Pattern in Zygomatic Implants Supporting Different Superstructure Materials

**DOI:** 10.3390/ma15144953

**Published:** 2022-07-16

**Authors:** Artak Heboyan, Roberto Lo Giudice, Les Kalman, Muhammad Sohail Zafar, João Paulo Mendes Tribst

**Affiliations:** 1 Department of Prosthodontics, Faculty of Stomatology, Yerevan State Medical University, Str. Koryun 2, Yerevan 0025, Armenia; heboyan.artak@gmail.com; 2 Deptartment of Clinical and Experimental Medicine, Messina University, 98100 Messina, Italy; roberto.logiudice@unime.it; 3 Schulich School of Medicine and Dentistry, Western University, 1151 Richmond Str., London, ON N6A 3K7, Canada; lkalman@uwo.ca; 4 Department of Restorative Dentistry, College of Dentistry, Taibah University, Al Madinah Al Munawwarah, Medina 41311, Saudi Arabia; drsohail_78@hotmail.com; 5 Department of Dental Materials, Islamic International Dental College, Riphah International University, Islamabad 44000, Pakistan; 6 Department of Dental Materials, Academic Centre for Dentistry Amsterdam (ACTA), University of Amsterdam and Vrije Universiteit Amsterdam, 1081 LA Amsterdam, The Netherlands

**Keywords:** dental implants, finite element analysis, dental materials, materials, prosthodontics

## Abstract

The aim of this study was to assess and compare the stress–strain pattern of zygomatic dental implants supporting different superstructures using 3D finite element analysis (FEA). A model of a tridimensional edentulous maxilla with four dental implants was designed using the computer-aided design (CAD) software. Two standard and two zygomatic implants were positioned to support the U-shaped bar superstructure. In the computer-aided engineering (CAE) software, different materials have been simulated for the superstructure: cobalt–chrome (CoCr) alloy, titanium alloy (Ti), zirconia (Zr), carbon-fiber polymers (CF) and polyetheretherketone (PEEK). An axial load of 500 N was applied in the posterior regions near the zygomatic implants. Considering the mechanical response of the bone tissue, all superstructure materials resulted in homogeneous strain and thus could reconstruct the edentulous maxilla. However, with the aim to reduce the stress in the zygomatic implants and prosthetic screws, stiffer materials, such Zr, CoCr and Ti, appeared to be a preferable option.

## 1. Introduction

Dental implants remain the ideal treatment option for supporting prosthetic restorations in patients with partial and/or complete edentulism [1]. Unfortunately, in the atrophic edentulous maxilla, poor bone quantity and quality may inhibit the use of conventional dental implants [2]. In certain situations, bone augmentation with biomaterials may be required to improve the bone volume, enabling the clinician to perform more conventional rehabilitation treatment options with standard dental implants [3,4]. However, in the case of severe maxillary atrophy, the use of zygomatic implants may be the only viable alternative available for full-arch rehabilitation, which may also reduce the requirement for bone graft procedures [2,5,6].

Traditional protocols recommended a healing phase, to allow for the proper osseointegration between the bone and implant [2,3]. During healing, the application of occlusal forces should be minimized [7]. Previous studies have indicated that dental implants could be immediately loaded without impacting the osseointegration phase [8,9,10]. This seems to be generally accepted as a clinical protocol for implant-supported, full-arch prostheses, in order to reduce the treatment time [6]. Conversely, implant-supported full-arch prosthesis designs require the splinting of a solid superstructure, that allows the transfer and appropriate distribution of the occlusal loads between the implants and supporting bone, limiting the amount of micromovements [2,7,11,12]. In terms of biomechanical behavior of the superstructure, various factors may affect the flexural strength and performance, including the thickness, cross-sectional design and structural materials [12,13,14].

With the application of CAD/CAM, there are several superstructure materials that are suggested for implant-supported, full-arch rehabilitations, such as a cobalt–chrome alloy [2], a titanium alloy [7], zirconia [15,16], carbon-fiber polymers [17,18] and the polyaryletherketone family [14,19]. Despite the extensive literature on the effects of superstructure materials for conventional implants [6,11,12,13,14,15,16,17,18,19,20,21], the data regarding biomechanical behavior of zygomatic implants are limited [2,6,21,22].

Both biological tissues and the stress/strain patterns of synthetic structures can be assessed using 3D finite element method (3D-FEM) [20]. Anatomically complex structures can be geometrically designed and correlated with the mechanical properties through finite element analysis. The calculated stress can also be evaluated to compare simulated models and to assist in determining if the planned oral rehabilitation could potentially fail mechanically under loading conditions.

The purpose of this current investigation was to determine the superstructure elastic modulus associated with zygomatic implants on the stress and strain under loading. The null hypothesis was that superstructure stiffness from different materials does not affect the mechanical behavior of zygomatic implants in maxillary rehabilitation.

## 2. Materials and Methods

**Figure 1:**
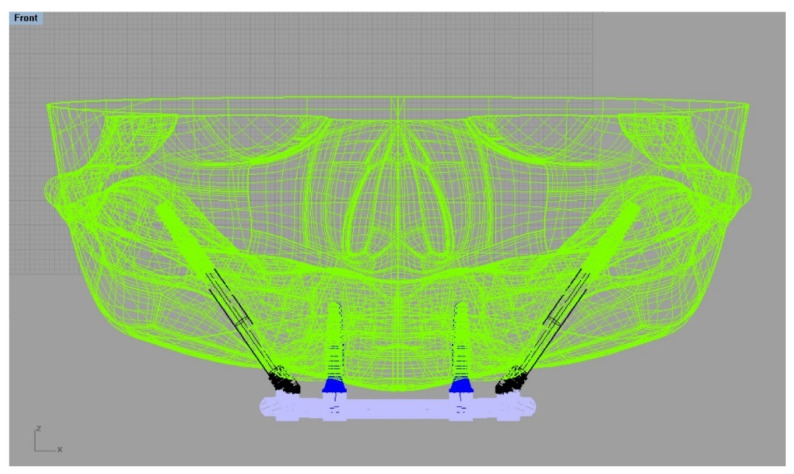
Polylines network in the CAD software created for three-dimensional modelling of a skull with fixation of the four implants. Anterior implants with conventional design and posterior implants as zygomatic implants.

A computer-generated model of a skull without the presence of anomalies was transferred in DICOM format from the database [23]. The 3D slicer software was used to convert the file to STL (Stereolithography). Using CAD software (Rhinoceros Version 4.0 SR8, McNeel North America, Seattle, WA, USA), a simulated model of an edentulous maxilla was designed, incorporating the major anatomical features of an adult human, including shape, size, and absence of abnormalities [23] (Figure 1).

**Figure 2:**
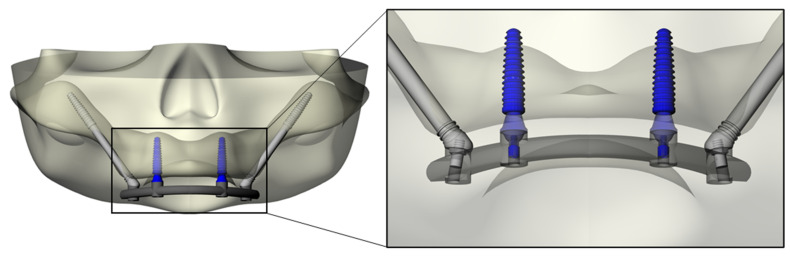
Three-dimensional simulated model developed for the present study. At higher magnification, it is possible to observe the implants, abutments, prosthetic screws and position of the superstructure.

The modeling step was performed following BioCad protocol to create the 3D volume model with solid characteristics [24]. For this simulation, the anatomical structure of the bone was designed with polylines, and the intersection of three/four polylines was joined to create the individual network surface. The union of intersected and joined regions was used to create the final 3D shape of the model. Sequentially, Morse-taper dental implants (10 × 4.1 mm) (Conexão Sistemas de Prótese, Arujá, Brazil) previously designed [14] were exported into the CAD and placed in the anterior portion of the maxilla, symmetrically near the canine regions [14]. The platform had a diameter of 4.1 mm. Mini-conical abutments, as well as their corresponding screws, were designed for each implant. Subsequently, zygomatic implants with a tilted head, monoblock conical form and with apical threads (3.6 × 33.5 mm) were designed and positioned bilaterally in the molar sites [2]. For the zygomatic implants, abutments were not used. The superstructure was designed following the contour of the maxilla (U) and the implant’s platform. This resulted in flat surfaces and rounded corners of 4 mm width and 3 mm maximum thickness [14] (Figure 2).

**Figure 3:**
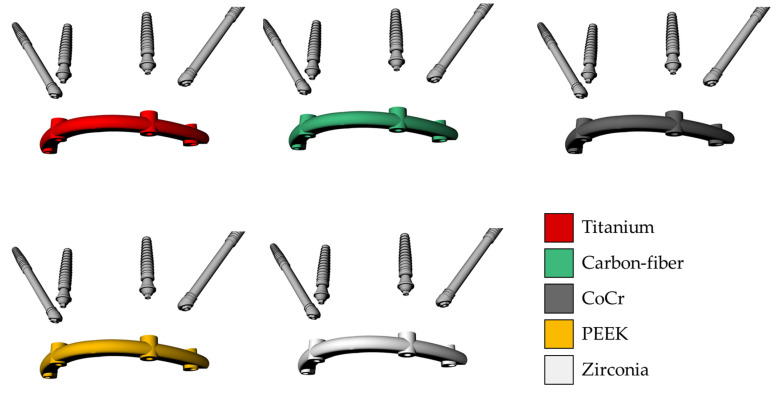
Schematic illustration of each simulated material for the superstructure in contact with the implants. Different colors represent different elastic moduli.

**Figure 4:**
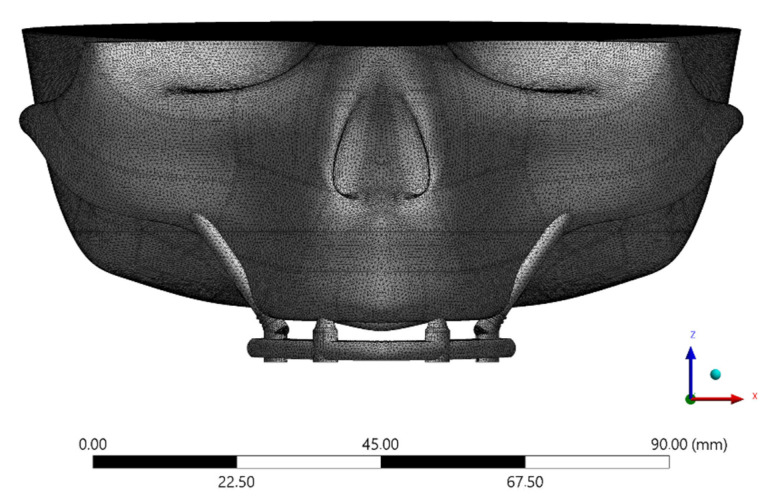
Meshing division created with tetrahedral elements during the pre-processing step.

The solid 3D geometries were then imported into the computer-aided engineering software (ANSYS 19.2, ANSYS Inc., Houston, TX, United States) in STEP format. The material’s constants (elastic modulus and Poisson ratio) were assigned to each volumetric element with isotropic and homogeneous consistency, based on data in the literature. The properties have been summarized in Table 1 [15,21,25,26,27,28]. Five different superstructures have been simulated, as showed in Figure 3. The implants and abutments were simulated with a titanium alloy (Table 1). The contacts were considered ideally bonded between the structures. A 3D mesh was generated subdividing the geometry into a finite number of elements. The elements considered were the tetrahedral type for all models. The total number of elements (808664) and nodes (1365383) for the final setup were determined by a convergence test (Figure 4).

For the boundary conditions, the top surface of the skull was limited in all directions and the force was symmetrically applied at the posterior surface of the superstructure. A load was applied in a circular region of 2 mm^2^ in the regions of the left and right first molars of 500 N [14]. The force exerted of 500 N is equal to the maximum force recorded by osteomatognatic units [29] as well as corresponding to a physiological average load for human bite force [30,31,32]. However, parafunctional loading could present axial and oblique loads simultaneously and with values higher than 1000 N [31].

The obtained data for the framework, abutments, implants, and screws were calculated in von Mises stress distribution as well as in microstrains (με/με) for bone tissue [7].

## 3. Results

**Figure 5:**
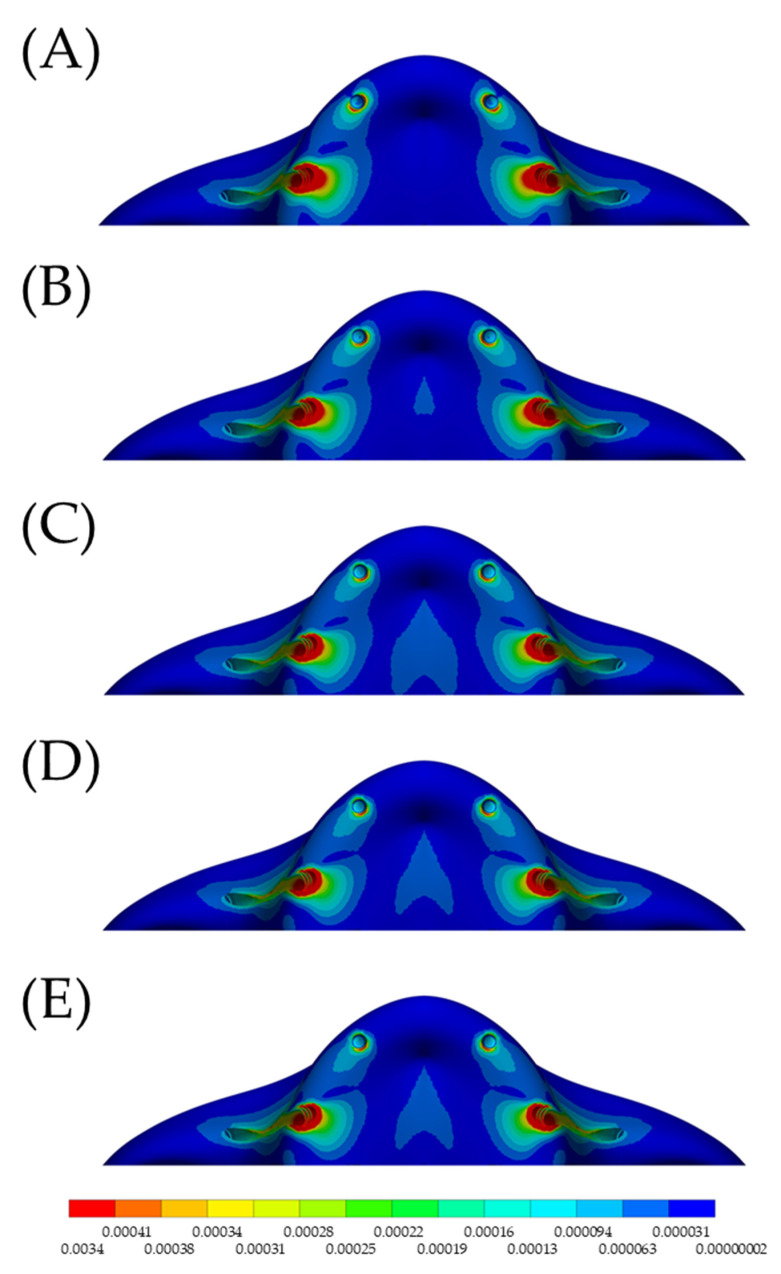
Occlusal view of the microstrain distribution in the maxillary bone according to the different superstructure materials. (**A**) PEEK, (**B**) CF, (**C**) Ti, (**D)** CoCr and (**E**) Zi. There is higher stress in the posterior implant regardless of the evaluated model.

**Figure 6:**
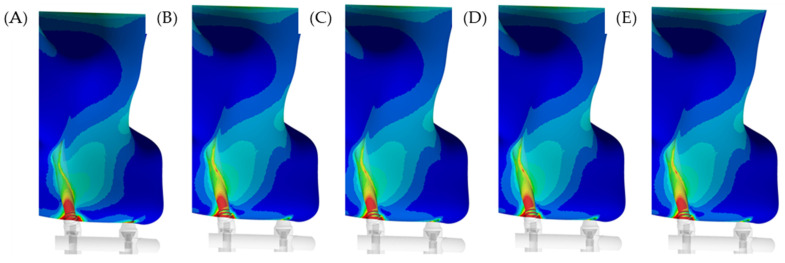
Lateral view of the microstrain distribution in the maxillary bone according to the different superstructure materials. (**A**) PEEK, (**B**) CF, (**C**) Ti, (**D**) CoCr and (**E**) Zi. In this view, the red area with high stress can be reduced as the superstructure stiffer increases.

In the maxilla, the microstrain pattern distribution, relevant to each superstructure biomaterial, was visible in colorimetric graphs in Figure 5 and Figure 6. Figure 5 illustrates the occlusal view, and depicts that the strain was higher in the posterior peri-implant region near the zygomatic implant platform. The stiffer models (zirconia and CoCr) displayed favorable mechanical response for the peri-implant bone, with lower strain. The microstrain criteria application, which is based on the biologic “machinery”, suggests that the entire bone strength formulate a tissue-level according to a negative feedback system, called the mechanostat. Therefore, regardless of the superstructure material, there are no values within the physiological limits of bone (>3000 and <500 με). In a lateral view (Figure 6), it is illustrated that the strain is distributed along the implant axis, even in the regions without threads.

Using the mechanical APDL auto-probe, it was possible to locate the highest stress value per structure in the model. The stress peak for the superstructure itself was proportional to the elastic modulus (Table 2). The higher the stress concentration in the superstructure, the lower the stress peak on the zygomatic implants and associated prosthetic screws. Conversely, with the conventional anterior implants, higher stress occurred with the simulated zirconia superstructure.

**Figure 7:**
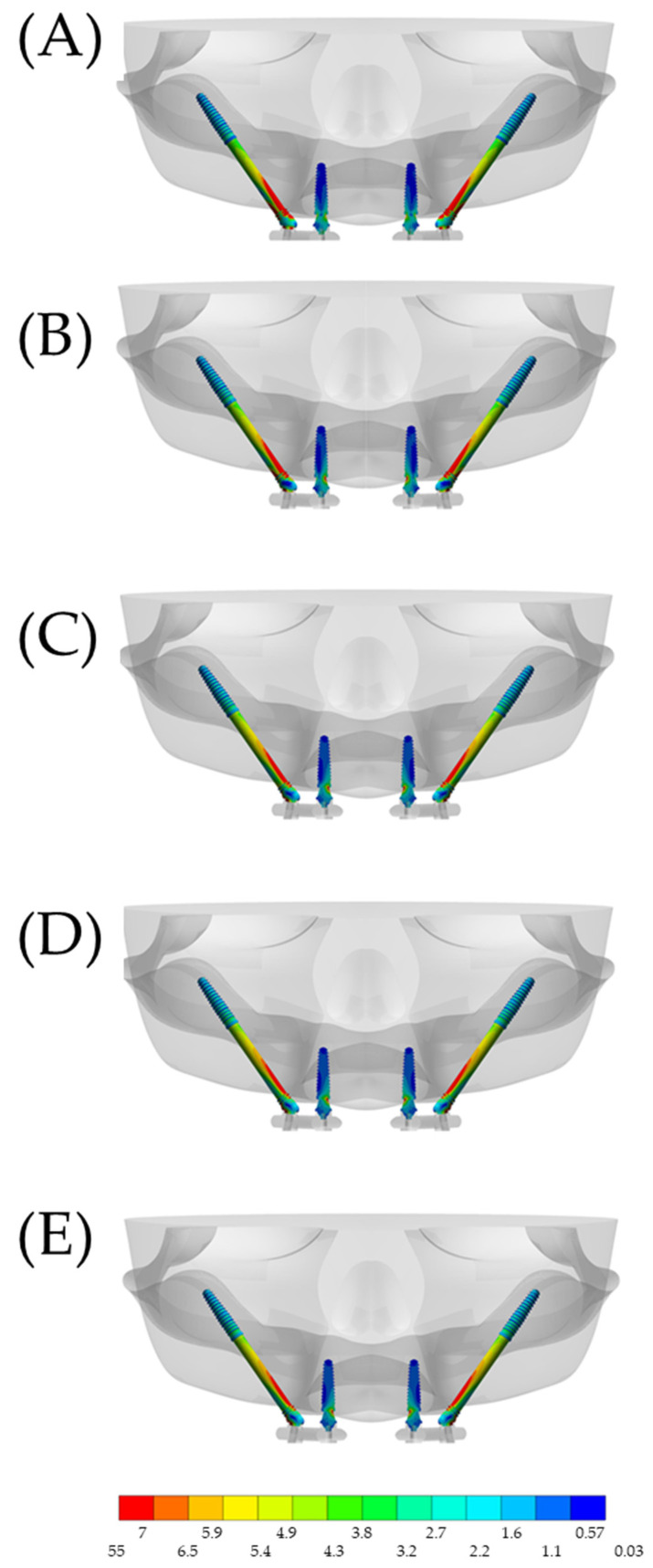
Sectioned frontal view (anterior implants axis) of the von-Mises stress distribution in the implants according to the different superstructure materials: (**A**) PEEK, (**B**) CF, (**C**) Ti, (**D**) CoCr and (**E**) Zi. For the anterior implant, the abutment–abutment joint is the most affected region, while the zygomatic implant concentrates stress along its structure.

The high stress concentration with the stiffer superstructures resulted in a lower stress distribution in the zygomatic implants, which mainly affected the most distal implants (Figure 7). The anterior implants showed little variation in stress concentrations between the models, when comparing the values in the posterior zygomatic implants.

When observing the section plane through the implant axis, it is possible to appreciate that the stress mainly affects the cervical portion, with minimal stress at the apex of the implant. This corresponds to the fulcrum around the bone level at the cervical region. For the anterior implants, the most damaged area was around the abutment neck.

**Figure 8:**
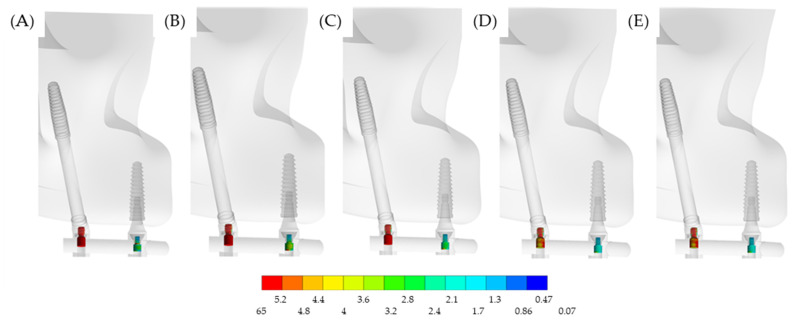
Lateral view of the von-Mises stress distribution in the prosthetic screws according to the different superstructure materials: (**A**) PEEK, (**B**) CF, (**C**) Ti, (**D**) CoCr and (**E**) Zi. Both posterior and anterior prosthetic screws were involved in the load distribution, with a greater effect at the posterior one. Regardless, when the elastic modulus of the superstructure material decreases, the stress concentration on the screw increases.

In a lateral view, Figure 8 illustrates the stress on the prosthetic screws. Evidently, the most affected screws were located in the posterior region, near the loading. However, the stress may be affected by the superstructure material.

## 4. Discussion

The present study used a CAD software to design a tridimensional model of an edentulous maxilla and evaluated the stress–strain pattern of zygomatic dental implants with different superstructures. The null hypothesis was that stiffness from different materials does not affect the mechanical behavior of zygomatic implants in maxillary rehabilitation. This was rejected since there was a difference in the stress, as related to each simulated condition.

Based on the present simulation, the higher strain in the posterior peri-implant tissue near the zygomatic implant platform seems to be adequate, as the zygomatic implants and corresponding bone are well suited. This supports the relatively high success rate of zygomatic implants (12-year cumulative survival rate of 95.21%) and reinforces the treatment option as a predictable approach, especially in patients with limited maxillary bone density [33,34]. This simulation supports that properly placed implants provide the most predictable outcome, in terms of stress–strain behavior, which is likely to influence the success of treatment. The placement of zygomatic implants near the molar region seems to be ideal and has been previously reported in the literature [35,36,37].

As the data illustrated, the higher the stress concentration in the superstructure, the lower the stress on the zygomatic implant and its corresponding prosthetic screw. Therefore, considering only the stress values, zirconia can be considered as a material of choice due to its role in limiting the stress on implants and screws. However, a previous literature review indicated that for implant-supported fixed dental prostheses, the conventionally bi-layered zirconia structure should not be considered as the material of choice, due to its high risk for fractures and chipping of the veneer material. Monolithic zirconia may be a promising alternative, but more clinical data are required [38].

Future research may explore 3D printed and additively manufactured superstructure materials, specifically a titanium alloy and zirconia, either in a solid form or in the newly explored lattice structure [36]. The lattice structure may provide a mechanical behavior that could be favorable for long-term success. Moreover, additive manufactured workflows have been explored that provide drastic improvements in efficiency and cost [36]. The fabrication pathway could also be delivered at the point of care, as a customized patient-specific prosthesis, dramatically improving the workflow. Therefore, the processing method may affect the accessibility and sustainability of this treatment modality and further studies are required to confirm this hypothesis.

In the maxilla, the pneumatization of the maxillary sinuses, centripetal alveolar resorption pattern, nasal fossae and nasopalatal duct may lead to severe atrophy [37]. The rehabilitation of the severe atrophic maxillary could include different techniques that have shown different success and survival rates [38,39,40,41,42,43,44]. In the present simulation, the modelling considered the zygoma anatomy-guided approach, considering a ZAGA type II with a combined extra- and intra-sinus path with most of the implant body being located extra-sinusally [45]. However, other conditions could modify the results presented and should be evaluated further [45,46,47,48,49].

To clarify the impact of implant surgery, a previous study evaluated and compared 3 zygomatic implantation approaches for the restoration of the extensively atrophied edentulous maxilla using a finite element method [47]. According to the authors, all simulated zygomatic implant systems resulted in homogeneous load distribution and thus could be used to rehabilitate the edentulous maxilla. However, the exteriorized method, with one dental implant in the lateral incisor area, seemed to be the most suitable reconstruction method for the severely resorbed edentulous maxilla [47]. That model is very similar to the model simulated in this investigation.

Guided Bone Regeneration (GBR) with titanium meshes [39,40], bone grafts [40,41] and sinus lifting surgeries [38,39,40,41,42] are associated with post-operative complications, long rehabilitative times and high cost. Zygomatic implants [43], splinted with conventional implants, may be considered an alternative to bone augmentation surgery in patients with a severely atrophic maxilla that prefer a fixed prosthetic rehabilitation that can be reliable, even with immediate loading. The present investigation supports this information, suggesting that a stiffer framework should be used for an improved stress distribution. The rehabilitation of patients with zygomatic implants, splinted with a superstructure, may be manufactured from different materials leading to a different pattern for the stress distribution. Since the 3D-FEM analysis is a reliable technique to evaluate the stress patterns comparing different materials [44], it was the methodologic approach applied in this study.

A previous finite element study evaluated the extent of micromotion of implants under immediate loading supported by titanium and CoCr superstructures [2]. The authors used a 3D model of a half-edentulous maxilla with a zygomatic posterior implant. Similar to the present study, they found that the greatest amount of micromotion occurred on the implant where the load was applied. However, they also found that the extent of micromotion was slightly higher in the implants with CoCr alloy superstructure. In the reported study, some factors influenced their results. The first was the absence of threads in the dental implants, which reduced the primary implant stability. Secondly, was the simulation of a non-linear condition with coefficient of friction between the surface of the implant and the neighboring bone [2].

Using numerical simulation, previous authors reported the effects of restorative materials and framework design on the stress within dental implants and peripheral bone [13]. According to the literature, the variations in the biomaterial and width of connectors may influence stress on cortical bone, cancellous bone, and implants. They also reported that cobalt-chromium-supported ceramic was related with the lowest stress values. These findings corroborate the present investigation, with similar mechanical behavior for superstructures supported by zygomatic implants.

According to an in vitro study, the mechanical response is an important issue in zygomatic implants that was not deeply investigated [46]. To overcome this, the authors investigated the mechanical strain in the supporting bone around zygomatic implants using photoelastic analysis. However, there are limitations inherent to the photoelastic analysis considering simplifications in bone complex and purely quantitative results [46]. Similarly, the present study presents limitations. The simulation was performed with linear-elastic behavior, there was no presence of different loading directions, parafunctional habits, oral biofilm, pH variation, fatigue effects, temperature variation, presence of vertical misfit between the superstructure and abutments, as well as antagonist teeth.

Another major limitation of the present investigation was the absence of numerical model validation using an in vitro experiment for the result’s reliability. Therefore, despite corroborating with previous investigations in terms of stress pattern and mechanical behavior, the results obtained should not be isolated or used to determine treatment reliability. Further investigations, considering photoelasticity, strain gauge or digital image correlation, could be performed to confirm or deny the differences observed in the present stress analysis [50,51]. In summary, the stress maps should be carefully evaluated before being extrapolated by further studies in dentistry.

## 5. Conclusions

Considering the mechanical response of bone tissue, all evaluated materials resulted in homogeneous strain and thus may be used to reconstruct the edentulous maxilla. However, with the aim to reduce the stress in the zygomatic implants and prosthetic screws, stiffer materials such as zirconia, CoCr and titanium, demonstrated a preferable mechanical behavior compared to the polymeric superstructures.

## Figures and Tables

**Table 1:** Mechanical properties simulated.

Structure	Elastic Modulus (GPa)	Poisson Ratio
PEEK	3.7	0.4
Titanium	110	0.3
CoCr	201	0.3
Zirconia	205	0.3
Carbon-reinforced polymer	42.7	0.3
Bone tissue	5600	0.28

**Table 2:** Stress peak calculated in each structure.

Material	Superstructure	Zygomatic Implant	Zygomatic Prosthetic Screw	Anterior Implant	Anterior Prosthetic Connection
PEEK	17.96	102.1	126.8	9.9	6.5
Titanium	27.07	55.24	64.5	13.7	8.8
CoCr	33.19	47.60	55.95	15.8	9.9
Zirconia	34.53	46.88	56.12	15.9	9.9
Carbon-reinforced polymer	25.12	72.95	80.65	10.8	8.1

## Data Availability

Not applicable.
